# High efficacy of the MACOP-B regimen in the treatment of adult Langerhans cell histiocytosis, a 20 year experience

**DOI:** 10.1186/s12885-015-1903-8

**Published:** 2015-11-09

**Authors:** Enrico Derenzini, Vittorio Stefoni, Cinzia Pellegrini, Letizia Gandolfi, Alessandro Broccoli, Beatrice Casadei, Federica Quirini, Lisa Argnani, Lorenzo Tonialini, Pier Luigi Zinzani

**Affiliations:** Department of Experimental, Diagnostic and Specialty Medicine- DIMES, Institute of Hematology and Medical Oncology L.A. Seragnoli, University of Bologna, Via Massarenti 9, 40138 Bologna, Italy

**Keywords:** Adult Langerhans cell histiocytosis, MACOP-B, Chemotherapy

## Abstract

**Background:**

Adult Langerhans cell histiocytosis (LCH) is an orphan disease. Chemotherapy is usually reserved to patients presenting with single system multifocal (SS-m) or multisystem (MS) disease but due to the lack of randomized studies no standard first line therapy has been defined yet. Pediatric regimens based on the vinblastine/prednisone backbone are not well tolerated in adults and probably less effective. We previously demonstrated high efficacy of the dose dense polichemotherapy regimen MACOP-B in 7 adult patients with SS-m or MS-LCH, in terms of high response rate and durable responses. Here we report an update of these data with the purpose of evaluating the long term efficacy of MACOP-B in adult LCH.

**Methods:**

Clinical data of all adult LCH patients (*n* = 17) diagnosed and treated at our Institution during the past 20-year period were retrospectively reviewed.

**Results:**

A total of 11 patients (6 with SS-m and 5 with MS-LCH) were treated with MACOP-B from 1995 to 2014. The overall response rate was confirmed to be 100 %, with a complete response of 73 % and a partial response rate of 27 %. Overall progression free survival was 64 %, and disease free survival after achievement of initial CR was 87 %. Overall survival rate was 82 % after 6.7 years of median follow-up.

**Conclusions:**

These data confirm high activity of MACOP-B in adult LCH, indicating that a substantial fraction of patients achieve long lasting responses and can be cured with this therapeutic approach.

## Background

Langerhans cell histiocytosis (LCH) is a rare, heterogeneous and potentially debilitating disease [1]. Highest incidence is observed among children with 2–10 cases per million, whereas in adults LCH affects only one or two cases per million and it is thus considered an orphan disease [1]. Due to its rarity and lack of prospective randomized trials there is no specific therapy for adult LCH and treatment schedules have been derived so far from pediatric protocols [2]. Chemotherapy is reserved to patients with single system multifocal (SS-m) or multisystem (MS) disease [3]. Since more than 25 years, the back bone of pediatric chemotherapeutic protocols is the combination of vinblastine and steroids, followed by therapy consolidation [3]. Given that early response emerged as an important prognostic predictor, efforts have been made to intensify the induction therapy by adding etoposide (in the LCH II trial) [4] or methotrexate (in the LCH III study) [5] to the standard vinblastine based regimen. These efforts were especially directed to treatment of patients with involvement of risk organs (RO). Nevertheless the results of these approaches were not deemed satisfactory, as no major differences in the final outcome were observed [4]. On the other hand the results of the LCH III trial suggested that milder induction but longer therapy duration could significantly improve the outcome by reducing recurrences [5]. Although with the 3 LCH trials substantial progresses have been made in the treatment of pediatric LCH, this therapeutic strategy is unlikely to be successful in adults, as the only prospective randomized trial evaluating the efficacy of vinblastine/prednisone regimen in adults (LCHA1 trial) was prematurely closed for unacceptable toxicities (vinblastine related neurotoxicity and detrimental effects of prolonged steroid therapy) [3]. Moreover recent studies seem to indicate that this approach might have lower efficacy in adults compared to children, suggesting that adult and pediatric LCH could harbor different biological characteristics [6, 7]. In conclusion, available data do not support the use of pediatric regimens in adult LCH, indicating that the concept of mild induction followed by maintenance therapy probably cannot be translated to adults. Alternative approaches tested in adult LCH include nucleoside analogs such as cytarabine and cladribine (2-CDA) [6, 8] but no consensus on the best frontline treatment strategy has been reached yet.

We previously described our experience with the dose dense short course polichemotherapy regimen MACOP-B (Methotrexate, Doxorubicin, Cyclophosphamide, Vincristine, Bleomycin, Prednisone) in 7 adult patients affected by SS-m or MS LCH [9], demonstrating high activity in terms of overall response rate (ORR) and long term disease control, despite the lack of maintenance therapy and an overall treatment duration of only 3 months.

Here we report the updated long term results of our experience with the MACOP-B regimen in the treatment of 11 adult LCH patients.

## Patients and methods

Data on 17 consecutive adult patients affected by LCH diagnosed and treated at our institution between 1995 and 2014 were retrospectively reviewed. For an institutional policy, in the absence of conclusive data and international guidelines on the best first line therapy, eligible patients with SS-m or MS-LCH were treated with the MACOP-B regimen. Eligibility criteria included age > 18 years, normal electrocardiography, adequate blood cell counts (white blood cells >3000/μL, hemoglobin > 10 g/dl, platelets > 100,000/ μL), normal liver and renal laboratory tests. Laboratory tests were repeated before the start of each chemotherapy cycle. All patients had biopsy proven diagnosis of LCH, and all biopsies were reviewed at our Institution. Staging procedures and response assessments and criteria were previously described [9]: briefly, complete response (CR) was defined as no evidence of active disease with regression of signs and symptoms at physical examination and imaging studies. A partial response (PR) was defined as a reduction of >50 % of all measurable and active disease. Initial staging included physical examination, complete endocrinological assessment, total-body computed tomography (CT) scan, complete skeletal X-ray, bone marrow biopsy and bone scan. All patients enrolled after the year 2002 (*n* = 8) underwent positron emission tomography (PET) scan. Patients with bone lesions were also evaluated with magnetic resonance imaging (MRI). Restaging was done after 6 weeks (interim evaluation) and 4 weeks after the completion of the last chemotherapy course with total-body CT scan, skeletal X-ray, MRI and bone scan in case of bone lesions and PET scan when feasible (8 patients).

Follow-up assessments were performed every 3 months during the first year and every 6 months starting from the third to the fifth year and every 12–18 months for the further follow-up.

**Fig. 1:**
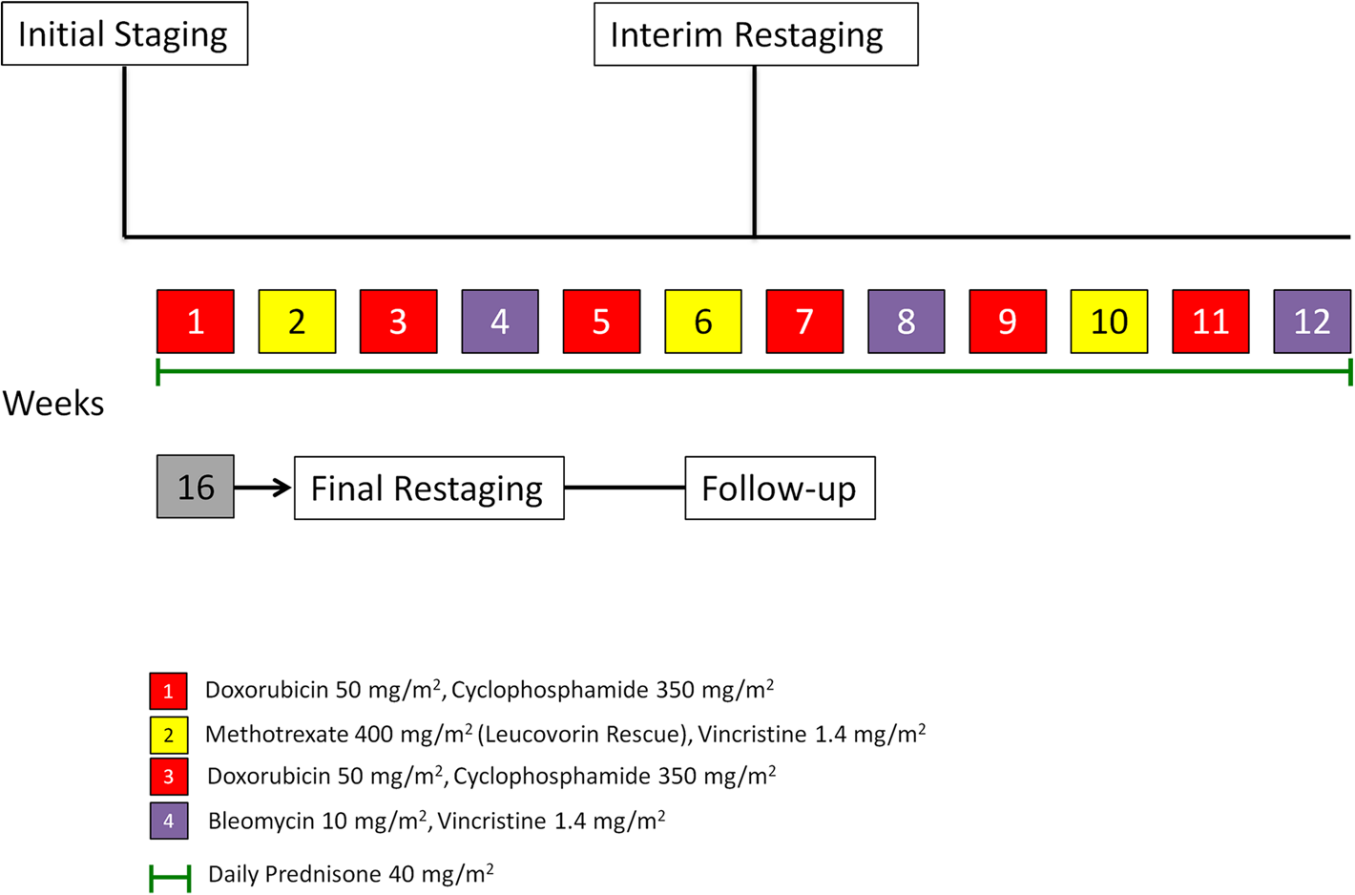
Treatment algorithm. Description of the treatment schedule and timing of staging procedures

MACOP-B regimen was administered weekly in outpatient basis for 12 weeks as reported previously [9]: cyclophosphamide 350 mg/m^2^ and doxorubicin 50 mg/mq given i.v. on days 1, 15, 29, 43, 57 and 71; methotrexate 400 mg/m^2^ on days 8, 36 and 64 followed by leucovorin rescue; vincristine 1.4 mg/m^2^ on days 8, 22, 36, 50 and 64; bleomycin 10 mg/m^2^ on days 22, 50 and 78 and prednisone 40 mg/m^2^ on days 1–84. The treatment algorithm is depicted in Fig. 1.

The study was approved by our institutional review board and by the Ethical Committee (Azienda Ospedaliera di Bologna, Policlinico S.Orsola-Malpighi) and registered in the Italian Registry of Observational Studies. All participants gave written informed consent in accordance with the Declaration of Helsinki.

Overall survival (OS) was calculated from the time of initial diagnosis until last follow-up or death. Progression free survival (PFS) was calculated from the start of chemotherapy until disease progression or last follow-up. Disease free survival (DFS) was calculated from the achievement of complete response after MACOP-B chemotherapy until relapse or last follow-up.

OS, PFS and DFS curves were analyzed with the Kaplan-Meier method [10].

## Results

From 1995 to 2014, 17 adult patients affected by LCH were treated at our institution. Four patients with monofocal LCH underwent local treatment strategies (surgery in 2 patients and radiotherapy in 2 patients). One patient with pulmonary LCH who relapsed after prior vinblastine/prednisone was monitored with a wait and see approach after smoking cessation. Of 12 patients initially diagnosed with SS-m (*n* = 6) or MS-LCH (*n* = 6), 11 were considered to be eligible for MACOP-B. One elderly MS-LCH patient with severe cardiologic comorbidities was deemed unfit for chemotherapy and treated with prednisone monotherapy. Characteristics of patients included in the study are shown in Table 1.

**Table 1:** General patients characteristics

Factor	N (%)
Number of patients	11
Age (median)	18–62 (40) years
Gender	
F	6 (55 %)
M	5 (45 %)
SS-m	6 (55 %)
MS	5 (45 %)
Risk organ involvement	5 (45 %)
Lung	4 (36 %)
Spleen	1 (9 %)
Prior therapy	2 (18 %)
Radiotherapy	1 (9 %)
Topical steroids	1 (9 %)

*N* number, *F* female, *M* male, *SS-m* single system multifocal, *MS* multisystem

**Fig. 2:**
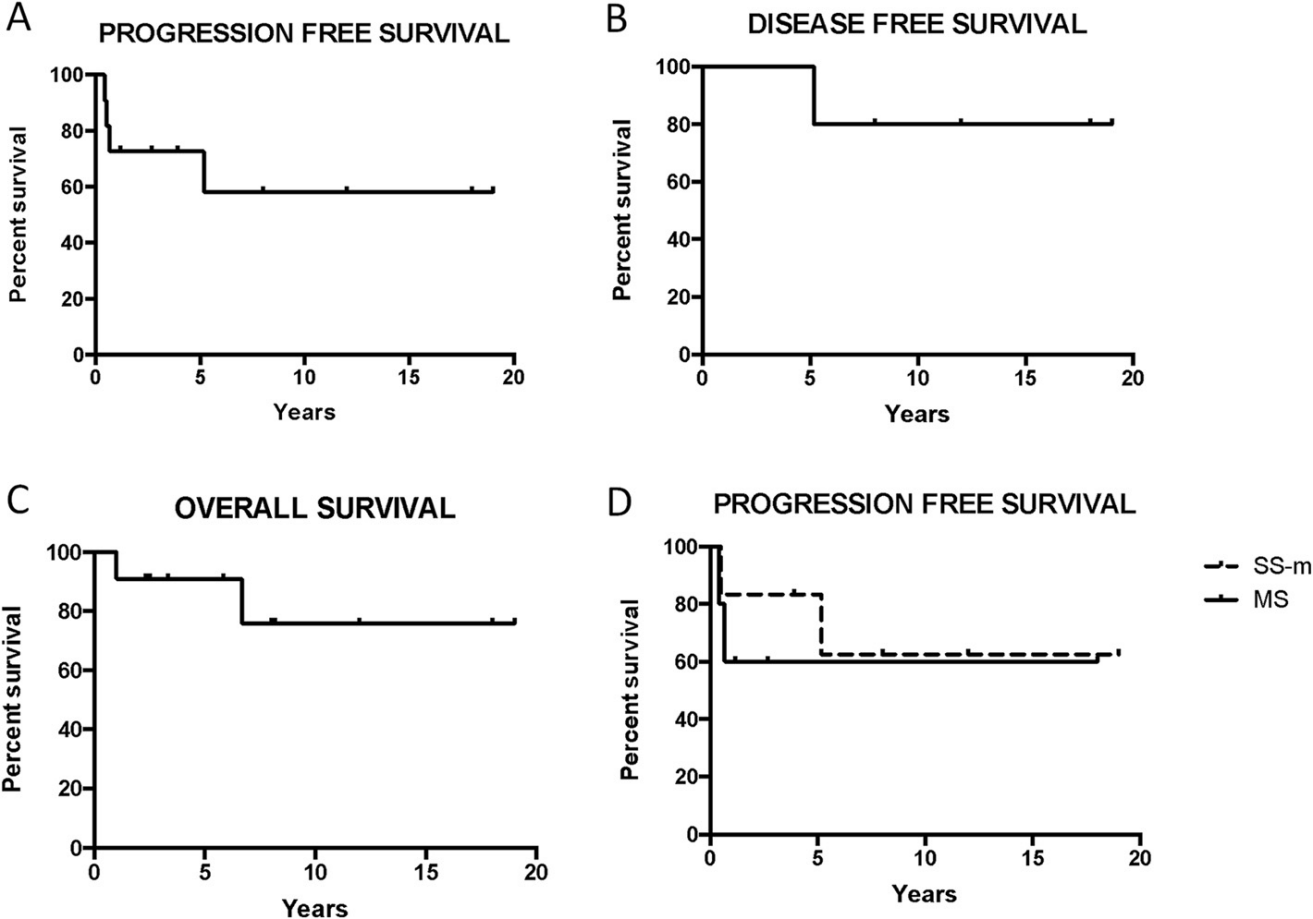
OS, PFS, DFS curves. **a** Progression-free survival of 11 adult LCH patients treated with the MACOP-B regimen in a 20-year period. **b** Disease-free survival of 8 adult LCH patients who obtained a CR after MACOP-B regimen. **c** Overall survival of 11 adult LCH patients treated with the MACOP-B regimen in a 20-year period. **d** Progression-free survival of 6 SS-m LCH and 5 MS LCH patients treated with the MACOP-B regimen in a 20-year period

All 11 patients treated with MACOP-B completed the planned 12 cycles and were assessable for response after 6 and 12 weeks. After 6 weeks overall response rate (ORR) was 100 % [6 CR (55 %) and 5 PR (45 %)]. After 12 weeks, at the final evaluation ORR was 100 % with 8 CR (73 %) and 3 PR (27 %). Four patients (36 %) (2 with MS, 2 with SS-m disease) relapsed or progressed after the achievement of initial response (1 after CR, 3 after PR), and overall PFS was 64 % (Fig. 2a). Notably after a median follow up of 6.7 years 7 of the 8 patients who initially obtained a CR are still in first continuous CR, with only 1 patient relapsed after 62 months, leading to a DFS rate of 87.5 % (Fig. 2b). All three patients (2 MS, 1 SS-m) who obtained a partial response progressed after 5, 6 and 8 months from the end of initial therapy. One MS-LCH patient underwent autologous stem cell transplantation (ASCT) after second line chemotherapy and is still disease free after 6 years from ASCT. Another SS-m patient relapsed after 6 months and is currently undergoing second line chemotherapy and salvage ASCT. The other 2 relapsed patients progressed and died of disease related complications, as reported previously [9]. OS rate was 82 % after a median follow-up of 6.7 years (2 deaths), and 8 of the 9 alive patients are disease free at the last follow up (after 228, 216, 144, 96, 66, 47, 32, 24 months of follow-up) (Fig. 2c). Detailed characteristics of single patients are described in Table 2.

**Table 2:** Detailed characteristics and outcome of the 11 LCH patients included in the study

Patient N°	Disease type (SS-m vs MS)	Response after MACOP-B	Relapse/Progression	DFS/PFS	Status
1	SS-m	CR	No	144+	Alive (CR)
2	MS	PR	Yes	8	Dead (PD)
3	SS-m	CR	Yes	62	Dead (PD)
4	MS	CR	No	228+	Alive (CR)
5	SS-m	CR	No	216+	Alive (CR)
6	MS	PR	Yes	5	Alive (II CR, +66 m after ASCT)
7	SS-m	CR	No	96+	Alive (CR)
8	MS	CR	No	47+	Alive (CR)
9	MS	CR	No	32+	Alive (CR)
10	SS-m	CR	No	24+	Alive (CR)
11	SS-m	PR	Yes	6	Alive (PD, ASCT ongoing)

*N* number, *SS-m* single system multifocal, *MS* multisystem, *CR* complete response, *PR* partial response, *PD* progressive disease, *DFS* disease-free survival, *PFS* progression-free survival, *m* months, *ASCT* autologous stem cell transplant

There was no difference in outcome (in terms of OS and PFS) between SS-m (*n* = 6) and MS-LCH patients (*n* = 5), with 2 of 5 MS-LCH patients who did not obtain a CR (3CR/2PR), compared to 1 of 6 SS-m patients (1PR/5CR). One patient died and 2 patients progressed or relapsed in both MS and SS-m groups (total 2 deaths, 3 progressions/1relapse) (Fig. 2d).

Overall, 8 patients were evaluated by PET scan. PET scan performed at initial diagnosis was negative in 2 patients, so that 6 patients were evaluated at week 6 and 1 month after the completion of MACOP-B. Interim PET performed at week 6 was negative in 4 of 6 patients, predicting final CR in 3 of 4 cases. Two patients converted from PR to CR from interim to final evaluation. Of 5 patients with negative post-therapy PET, 4 are still in first continuous CR.

Toxicities were mild and reversible, in line with previously published data on MACOP-B regimen in adult patients [9, 11]. Briefly, 4 patients had treatment delay due to grade 3 neutropenia, which was prevented by administering prophylactic granulocyte-colony stimulating factors (G-CSF) in subsequent cycles. Antibiotic prophylaxis with twice-weekly sulfamethoxazole-trimethoprim was given to all patients. Overall, no episodes of febrile neutropenia or serious infections were observed. Grade 3 self-limiting hypertransaminasemia was observed in one patient after methotrexate administration, which resolved in 1 week and was prevented by a 30 % dose reduction in the subsequent methotrexate cycles. No patient required treatment discontinuation.

## Discussion

In this report we present the updated long term results of our experience with the MACOP-B regimen in the treatment of adult SS-m and MS-LCH. With a CR rate of 73 %, an OS rate of 82 % and a PFS rate of 64 % these data confirm that MACOP-B is very effective in adult LCH, inducing long term complete responses in a significant fraction of patients. Regarding overall efficacy, these data are in line with the results of the LCH-III pediatric trial [5], but it should be noted that the duration of therapy is about 4-fold longer in the LCH-III pediatric protocol (12 months vs 3 months). As 87 % of patients obtaining a CR after MACOP-B are still disease free after a median follow-up of 6.7 years, these data indicate that long term disease control is achievable in adults without maintenance therapy, and highlight the curative potential of this therapy. These data reinforce the concept that the primary therapeutic aim in adult LCH should be the quality of initial response rather than long term maintenance to prevent recurrences. Moreover, the application of pediatric protocols in adults is generally difficult due poor tolerance, and in fact the LCHA1 trial was closed prematurely due to unacceptable toxicity [3]. Furthermore, multiple studies recently reported suboptimal efficacy of pediatric approaches in adults [3, 7]. The high efficacy of MACOP-B may also suggest a role for antracyclines and cyclophosphamide in adult LCH, in line with previous reports investigating dose dense regimens [12, 13], and with anecdotal case reports suggesting efficacy of antracycline based regimens in case of lymphomas or leukemias coexisting with LCH [14, 15]. Our data also compare favorably with recently published data with front line 2-CdA [7], which is widely used in adults. Regarding the role of PET scan, these results suggest that this imaging modality should be part of the diagnostic workflow of adult LCH patients, since the majority of our patients (6 of 8 patients) were PET positive at the moment of initial diagnosis. Although larger studies will be needed to assess the value of PET in the setting of response evaluation, the fact that 4 of 5 patients with negative post-therapy PET are in first continuous CR supports further evaluation of PET scan also in this context.

## Conclusions

In summary, in this report we confirmed high efficacy of MACOP-B regimen in adult SS-m or MS-LCH. The quality of initial response seems to be an important determinant of the final outcome, as most patients obtaining initial CR, achieve long-term remission and are eventually cured with this approach. However due to the retrospective nature of this study and to the small sample size we could not analyze the differential efficacy of this regimen in SS-m vs MS-LCH and RO+ vs RO- patients, and future efforts should be aimed at building risk adapted treatment algorithms, as a result of prospective randomized trials. Our understanding of the biology of LCH is rapidly improving and new druggable targets such as BRAF mutations [16] were recently identified. Vemurafenib showed preliminary evidence of efficacy [17], but no data are available yet on the long term efficacy of this therapy in adult LCH. In conclusion, we believe that the findings of this study provide the proof of principle for further testing of dose intense regimens in adult LCH, as treatment intensity and quality of initial response rather than treatment duration or maintenance therapy emerged as important determinants of the final outcome.

## Abbreviations

2-CDA: Cladribine
ASCT: Autologous stem cell transplantation
CR: Complete response
CT: Computed tomography
DFS: Disease free survival
G-CSF: Granulocyte-colony stimulating factors
LCH: Langerhans cell histiocytosis
MACOP-B: Methotrexate, Doxorubicin, Cyclophosphamide, Vincristine, Bleomycin, Prednisone
MRI: Magneting resonance imaging
MS: Multisystem
ORR: Overall response rate
OS: Overall survival
PET: Positron emission tomography
PFS: Progression free survival
PR: Partial response
RO: Risk organ
SS-m: Single system multifocal
